# Effect of Rotating Magnetic Field on Microstructure and Mechanical Properties of Cu-6Ni-6Sn Alloy

**DOI:** 10.3390/ma19204432

**Published:** 2026-10-21

**Authors:** Pengfei Wang, Yinghui Wei, Fengjie Lv, Shengli Chai, Ting Wu, Wenxin Fan, Zhongtao Zhang, Jinchuan Jie

**Affiliations:** 1 School of Mechanical Engineering, North University of China, Taiyuan 030051, China; 2 Northern Copper Co., Ltd., Yuncheng 043700, China; 3 Shanxi North Copper New Material Technology Co., Ltd., Yuncheng 043700, China; 4 Shanxi Key Laboratory of Copper-Based New Materials, Yuncheng 043700, China; 5 Golden Dragon Group Co., Ltd., Chongqing 404000, China; 6 School of Material Science and Engineering, Dalian University of Technology, Dalian 116024, China

**Keywords:** Cu-Ni-Sn alloy, rotating magnetic field, solidification microstructure, numerical simulation, strengthening mechanism

## Abstract

In this paper, the effects of rotating magnetic field (RMF) excitation current intensity on Sn segregation, grain refinement, and mechanical properties of Cu-6Ni-6Sn alloy were systematically investigated. The results demonstrate that the application of RMF can effectively refine the as-cast microstructure and mitigate Sn macro-segregation. The average grain size of the as-cast Cu-6Ni-6Sn alloy decreases monotonously with the increase in the excitation current, from 1563 ± 502 μm at 0 A to 207 ± 112 μm at 120 A. In addition, it was found that the difference in Sn content in the radial direction of the ingot reduced from 3.9 wt.% to 1.0 wt.%. The RMF improves heat transfer through forced convection, reduces temperature gradients and alleviates solute enrichment. It increases both thermal undercooling and the growth restriction factor to hinder grain growth and reduces constitutional undercooling to suppress segregation. In addition, the application of the RMF can synergistically increase the tensile strength and the elongation of the as-cast Cu-6Ni-6Sn alloy, with the values changing from 349 ± 4 MPa and 36.9 ± 0.8% at 0 A to 413 ± 4 MPa and 42.8 ± 0.9% at 80 A, respectively. This work quantitatively reveals the coupling relationship between RMF excitation current, grain refinement degree and Sn macro-segregation homogenization in Cu-6Ni-6Sn alloy, and quantitatively clarifies the dominant grain-boundary strengthening mechanism of the alloy under RMF.

## 1. Introduction

As a typical precipitation-strengthened copper alloy, Cu-Ni-Sn alloy has high strength, high elasticity, non-toxicity, excellent corrosion resistance and stress relaxation resistance [1,2]. It is regarded as an ideal system to replace beryllium bronze and has been widely used in aerospace, electronic information, petrochemical and mechanical engineering fields [3,4]. Among them, Cu-6Ni-6Sn alloy has important application value in high-end bearings and heavy machinery due to its outstanding wear resistance and excellent corrosion resistance [5]. However, the research on the strengthening mechanism, preparation method and multi-physical field simulation and regulation of the alloy is not sufficient, which leads to the strength-ductility matching of the alloy still needing to be improved. In recent years, the strategy of improving the comprehensive properties of Cu-Ni-Sn alloys by adding trace alloying elements has attracted wide attention. Zhang et al. [6] studied the effect of Mo content on the microstructure and properties of Cu-15Ni-8Sn alloy. It was found that an appropriate amount of Mo could inhibit discontinuous precipitation, improve composition segregation and increase hardness. The peak hardness of the 0.9 wt.% Mo alloy reached 384 HV after aging at 400 °C for 4 h. Yang et al. [7,8] showed that the addition of Nb can significantly refine the grains of Cu-15Ni-8Sn alloy and form the NbNi_3_ phase to inhibit discontinuous precipitation. The tensile strength of the alloy can reach 725 MPa and the over-aging stability is greatly improved for Cu-15Ni-8Sn-0.2Nb alloy. Zhou et al. [9] investigated the effect of Mn content on the microstructure and properties of as-cast Cu-9Ni-6Sn alloy. The results showed that 0.1 wt.% Mn can transform columnar crystals into equiaxed crystals, refine grains and improve Sn segregation. Meanwhile, the introduction of Mn increases the undercooling of the alloy and reduces the nucleation radius. Xie et al. [10] investigated the influence of adding 0.6 wt.% Si on the solidification behavior and microstructural evolution of a Cu-15Ni-8Sn alloy. The results demonstrated that the introduction of Si lowers both the liquidus temperature and the eutectic temperature associated with the γ_1_ phase and promotes the formation of Ni_31_Si_12_ intermetallic precipitates, which completely suppresses the development of the α/γ lamellar structure while concurrently refining the dendritic morphology. Lu et al. [11] incorporated a small amount of rare-earth Y into the Cu-12.5Ni-5Sn alloy, leading to the formation of nanoscale Ni_3_Y precipitates. These precipitates serve as effective heterogeneous nucleation sites for discontinuous precipitation and simultaneously refine the grain structure. As a result, they suppress discontinuous precipitation during aging, thereby significantly enhancing both the hardness and yield strength of the alloy. Zhang [12] and Lu [13] demonstrated that the addition of trace Zr element significantly refines the as-cast grain size of the Cu-15Ni-8Sn alloy and promotes the formation of nanoscale Ni_5_Zr and Ni_4_SnZr intermetallic precipitates. These precipitates effectively suppress discontinuous precipitation and induce a morphological transition of the γ phase from lamellar to acicular or granular. At a Zr content of 0.5 wt.%, the tensile strength of the Cu-15Ni-8Sn alloy reaches 980 MPa. So far, enhancing the microstructural homogeneity and mechanical performance of Cu-Ni-Sn alloys has predominantly relied on trace-element alloying. However, the introduction of additional alloying elements may reduce the chemical purity of the alloy and lead to adverse side effects. Therefore, a simple and non-destructive method to control the microstructure and mechanical properties of high-performance Cu-Ni-Sn alloys is still very necessary.

Electromagnetic stirring employs an electromagnetic field to induce forced convection in molten metals. Luo et al. [14] applied a downward traveling magnetic field (TMF) during directional solidification to tailor the solidification behavior of Al-Cu alloys exhibiting a broad solidification temperature range. The TMF-induced directional convection enhances solute transport and thermal homogenization while simultaneously refining dendritic morphology. Liu et al. [15] reported that a pulsed magnetic field (PMF) induces forced convection during the directional solidification of commercially pure aluminum. Upon PMF removal, suspended nuclei settle under gravity, the thermal gradient is re-established and a three-layer solidification structure forms. In this context, the application of an RMF offers a promising alternative; however, its systematic effects on the solidification behavior, Sn segregation, and mechanical properties of Cu-6Ni-6Sn alloys have not yet been established. This gap motivates the present investigation.

Compared with other magnetic field modes, the RMF induces uniform circumferential convection in the melt and produces a more stable flow field. Zhang et al. [16] showed that RMF can significantly promote dendrite fragmentation and refine solidification structure by inducing forced convection of melt. Liu et al. [17] reported that RMF application refines the brass matrix and promotes the morphological transformation of the NiAl(Si) phase from blocky to spherical. El-Taher et al. [18] reported that applying RMF reduced both the mushy zone and the melting point of Sn-Ag-Cu alloy, thereby improving its creep resistance and extending the creep rupture time. Li et al. [19] further demonstrated that RMF processing significantly refined the as-cast microstructure of Cu-8Sn-0.1P alloy, which is characterized by a wide mushy zone during solidification. Consequently, the average grain size decreased from 1639 μm to 52 μm. Under the optimal current of 120 A, the alloy exhibited significantly improved mechanical properties, with tensile strength, yield strength, and elongation reaching 389 MPa, 193 MPa, and 71.1%, respectively. These results indicate simultaneous improvements in both strength and ductility.

To address the research gap identified above, this study systematically investigates the effects of an RMF on the solidification microstructure and mechanical properties of Cu-6Ni-6Sn alloys. The originality of this work lies in the systematic application of RMF to the solidification of these alloys, the establishment of quantitative correlations among excitation current, grain size, Sn segregation extent, and mechanical properties, and the integration of experimental characterization with finite-element simulations to elucidate the underlying mechanisms governing grain refinement, suppression of segregation, and the synergy between strength and ductility. The main contribution is to demonstrate that RMF can effectively refine the as-cast microstructure, mitigate Sn macro-segregation, and simultaneously improve both tensile strength and elongation without the need for additional alloying elements.

## 2. Materials and Methods

### 2.1. Sample Preparation

**Figure 1:**
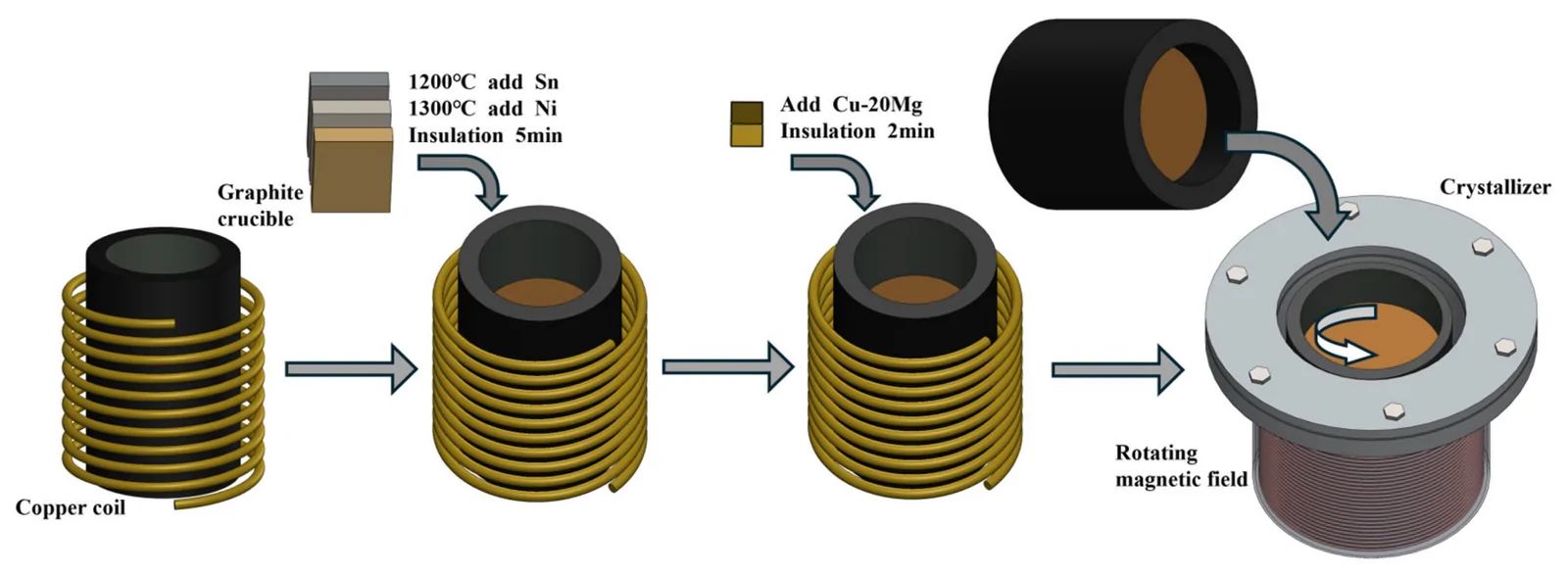
Schematic diagram of the alloy preparation process.

Alloy melting was carried out using a GP-100 medium-frequency induction furnace (Yiphee Electric Furnace Co., Ltd., Foshan, China). During solidification, a custom RMF generator with a frequency range of 0–20 Hz and a current range of 0–120 A was applied. High-purity raw materials were used, including electrolytic copper (≥99.99 wt.%), electrolytic nickel (≥99.95 wt.%) and industrial-grade pure tin (≥99.9 wt.%). According to the designed Cu-6Ni-6Sn composition (6.0 wt.% Ni, 6.0 wt.% Sn, balance Cu), electrolytic copper was first melted in a crucible, and a 3–5 mm layer of charcoal powder was added to the melt surface as a protective covering. When the melt temperature reached 1200 °C, industrial pure tin was added, followed by electrolytic nickel at 1300 °C. The melt was gently stirred to ensure uniform composition and held at 1300 °C for 5 min. A Cu-20Mg master alloy was then added and held for an additional 2 min after melting. After removing the surface slag, the melt was poured into a crucible preheated to 700 °C within the RMF system. The casting temperature was maintained at 1300 °C, while the RMF frequency was set to 20 Hz and the excitation current was varied at 0 A, 40 A, 80 A and 120 A. The frequency of 20 Hz was selected based on preliminary trials showing that lower frequencies (<10 Hz) produced insufficient melt convection, while higher frequencies (>30 Hz) led to excessive surface turbulence without additional refinement benefits. The alloy preparation workflow is shown in Figure 1. Standard specimens for microstructural characterization and property testing were then machined. Samples were collected 10 mm from the edge of the ingot, and the chemical composition of the as-cast ingots was determined by X-ray fluorescence (XRF), as summarized in Table 1.

### 2.2. Microstructure Analysis

The phase composition of the alloys was analyzed using a D8 Advance X-ray diffractometer (XRD). The as-cast microstructures of the Cu-6Ni-6Sn alloys were characterized using an Axio Observer A1 optical microscope (OM; Olympus BX51) and scanning electron microscopes (SEM; Thermo Fisher Verios 5 uc + symmetry). Prior to OM and SEM observations, the specimens were ground with abrasive papers and then mechanically polished. Secondary electron imaging was conducted at an accelerating voltage of 10 kV. The samples were etched using a solution composed of 2 mL HCl, 96 mL ethanol and 3 g FeCl_3_.

### 2.3. Mechanical Characterization

**Figure 2:**
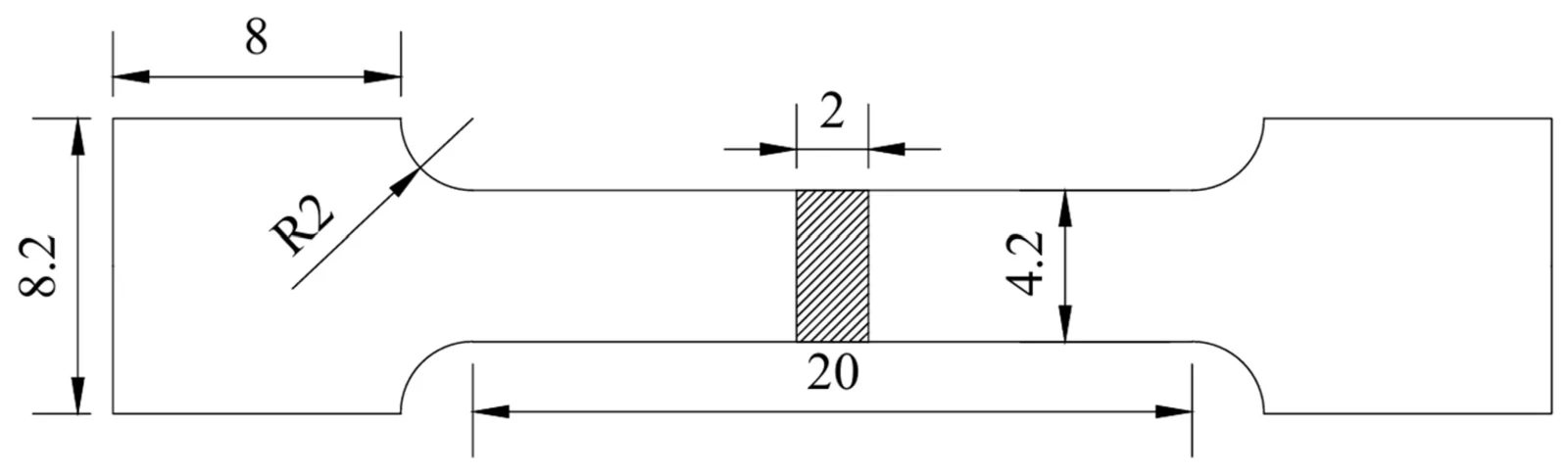
Schematic diagram of tensile specimen (unit: mm).

The mechanical properties of the as-cast samples were evaluated by uniaxial tensile tests at room temperature using a UTM 5105 tensile testing machine. The tests were performed at a constant tensile rate of 0.6 mm/min. The schematic diagram and dimensions of the tensile specimens are shown in Figure 2. To ensure the reliability and reproducibility of the results, three specimens were tested under each condition, and the average values were reported. In addition, the fracture morphologies of the tested specimens were examined using SEM.

### 2.4. Multiphysics Field Simulation

To investigate the effects of RMF on the solidification behavior of Cu-6Ni-6Sn alloys, a Multiphysics simulation was conducted. The solidification process under RMF influence was modeled using COMSOL Multiphysics 6.1 (COMSOL, Inc., Burlington, MA, USA). The magnetic field enhances melt flow by generating electromagnetic forces within the conductive melt. After the RMF is applied, the electromagnetic force is applied to the conductive melt to improve the flow of the melt. Therefore, the Lorentz force and flow field characteristics are mainly discussed.

(a) Modeling Parameters

The RMF stirrer used in the experiment has a height of 450 mm, with inner and outer diameters of 226 mm and 515 mm, respectively. The graphite crucible has a height of 300 mm, with an inner diameter of 60 mm, an outer diameter of 80 mm, and a depth of 280 mm, and its base is aligned with the bottom of the stirrer. The prepared 3 kg Cu-6Ni-6Sn molten alloy yields a melt height of 200 mm. The stirrer adopts a six-pole concentrated winding configuration, and the entire casting process was performed at a fixed temperature of 1300 °C.

(b) Geometric Model

**Figure 3:**
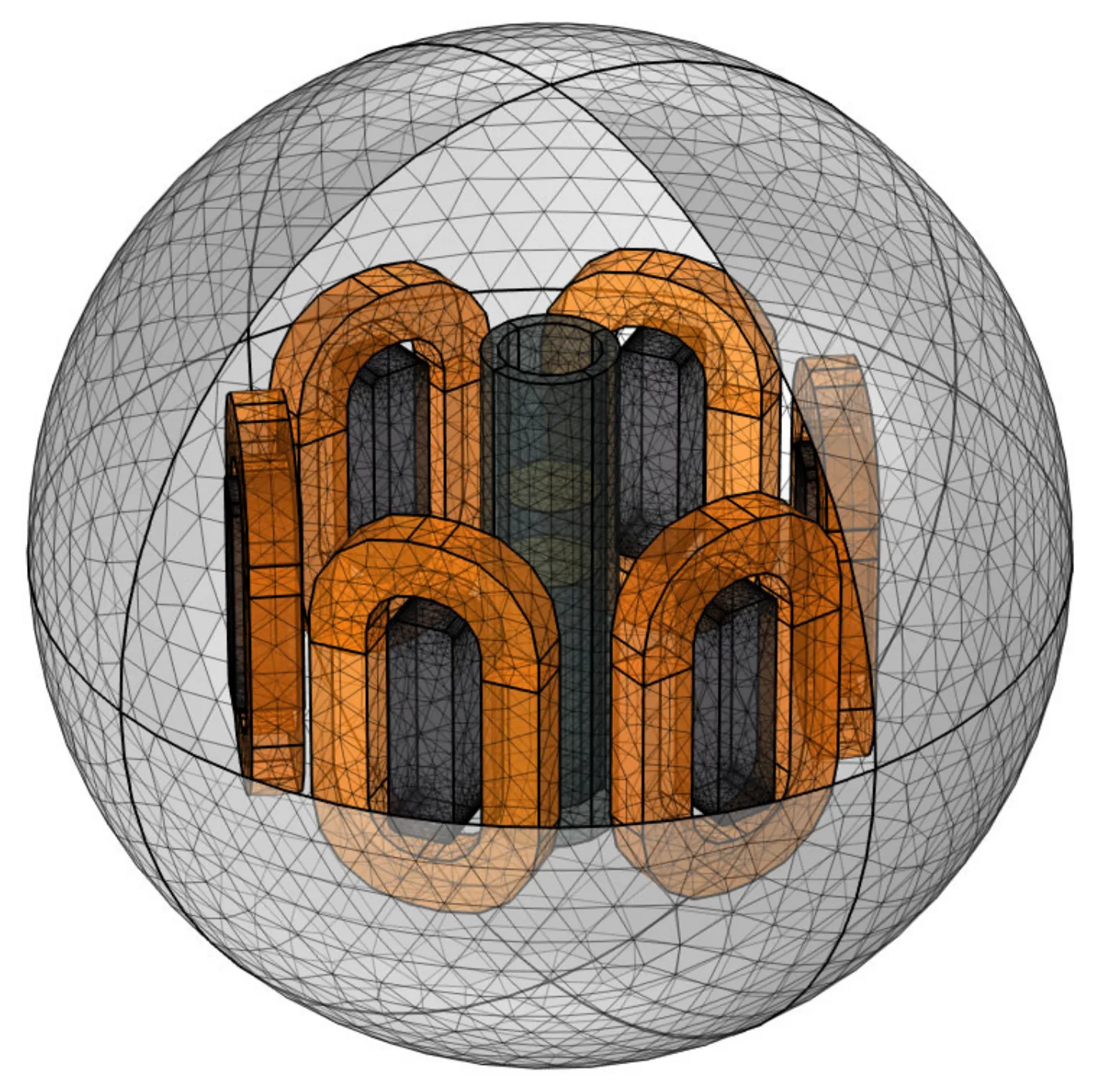
Geometric model of the RMF system.

A three-dimensional (3D) geometric model was constructed at a 1:1 scale based on the experimental apparatus, as shown in Figure 3. The computational domain was discretized using a tetrahedral mesh, generating a total of 329,680 elements.

(c) Governing Equations

An electromagnetic model was constructed to describe the solidification behavior of the Cu-6Ni-6Sn alloy under the RMF. The magnetic field generated by the six-coil concentrated winding stirrer was described using Maxwell’s equations, which consist of four governing laws: the Ampere–Maxwell law, relating time-varying electric fields to magnetic fields; Faraday’s law, relating time-varying magnetic fields to electric fields; Gauss’s law for magnetism, describing static magnetic fields; and Gauss’s law for electricity, describing static electric fields. The differential forms of these equations are given below [20]:

(1) $$\nabla \times H=J+\frac{\partial D}{\partial t}$$

(2) $$\nabla \times E=-\frac{\partial B}{\partial t}$$

(3) ∇⋅B=0

(4) ∇⋅D=0

where *E* is the electric field intensity (V/m), *B* is the magnetic flux density (T), *H* is the magnetic field intensity (A/m), *J* is the current density (A/m^2^), and *D* is the electric displacement vector (C/m^2^). The physical implications of the governing equations are illustrated as follows. Equation (1) reveals that the curl of the magnetic field is generated by conduction current and displacement current. Equation (2) demonstrates that a time-varying magnetic field acts as the curl source of the electric field. Equation (3) indicates that the magnetic field is divergence-free. Equation (4) shows that the divergence of the electric field is determined by electric charge.

In addition, the constitutive equations describing the electromagnetic properties are provided below [20]:

(5) $$D=\varepsilon_{0}\varepsilon_{r}E$$

(6) $$B=\mu_{0}\mu_{r}H$$

(7) J=σE

where *ε*_0_ is the vacuum permittivity (8.85 × 10^−12^ F/m), *ε*_r_ is the relative permittivity, *μ*_0_ is the vacuum permeability (4π × 10^−7^ H/m), *μ_r_* is the relative permeability and *σ* is the electrical conductivity (S/m).

The electromagnetic force acting on the melt can be calculated as follows [21]:

(8) F=J×B

where *F* represents the Lorentz force per unit volume (N/m^3^). The flow field of the incompressible melt is described by the continuity equation and the Navier–Stokes equations [22]:

(9) $$\rho \frac{\partial u}{\partial t}+\rho u\cdot \nabla u=-\nabla P+\mu \nabla^{2}u+F$$

where *μ* is the dynamic viscosity (Pa⋅s), *ρ* is the density (kg/m^3^) and *u* is the melt velocity (m/s).

(d) Initial Conditions

The initial temperature was set to the casting temperature of 1300 °C. The initial velocity field was defined with a downward vertical velocity of 70 mm/s, while velocities in other directions were set to zero. The initial magnetic vector potential was set to zero in all directions.

(e) Boundary Conditions

Heat transfer at the melt interface is described by the interfacial heat flux [23]:

(10) $$q_{0}=h(T_{ext}-T)$$

where *q*_0_ represents the interfacial heat flux (J/(m^2^·s)), *h* is the heat transfer coefficient (W/(m^2^·K)) and *T_ext_* is the external temperature (K). The RMF input consists of three-phase sinusoidal alternating currents, each phase shifted by 120°. The current densities for each phase are defined as follows [24]:

(11) $$I_{A}=I_{0}\sin(2\pi ft)$$

(12) $$I_{B}=I_{0}\sin(2\pi ft-\frac{2}{3}\pi )$$

(13) $$I_{C}=I_{0}\sin(2\pi ft+\frac{2}{3}\pi )$$

where *I*_0_ is the amplitude of the coil current density (A/m^2^); *f* is the frequency of the alternating current (Hz) and *t* is time(s).

(f) Model Assumptions

To simplify the Multiphysics coupling in the model, the following assumptions were made:

1. The copper melt is treated as an incompressible fluid.
2. Joule heating caused by the electromagnetic field is neglected due to the low current frequency.
3. The melt is assumed to satisfy the low-frequency approximation, so displacement currents are ignored.
4. Electromagnetic coils are modeled as current-carrying domains using a homogenized multi-turn coil interface.

(g) Physical Parameters

The materials, including the melt, coils, and iron core, are considered isotropic, with physical properties such as electrical conductivity and relative permeability treated as constant values, as summarized in Table 2.

The thermophysical properties of the materials adopted in the model were sourced from the COMSOL Multiphysics material library, with the main parameters summarized in Table 3.

## 3. Results

### 3.1. Phase Composition of As-Cast Specimens

**Figure 4:**
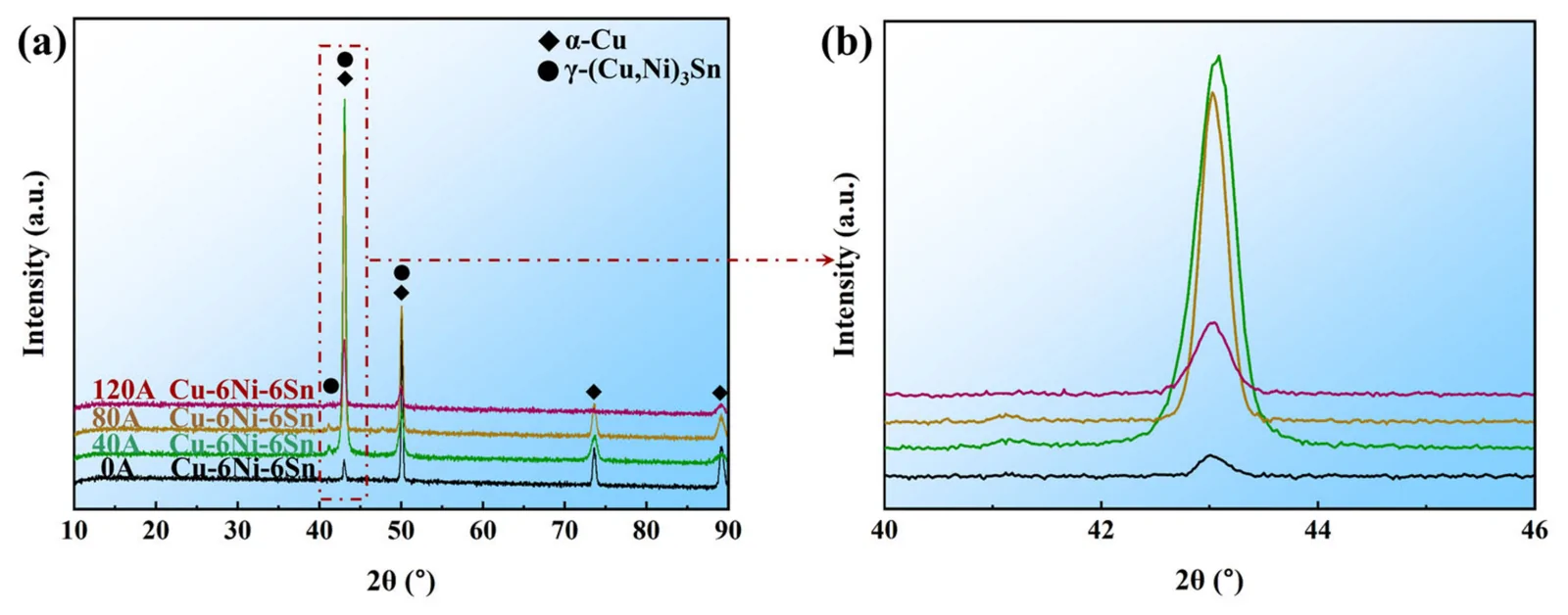
XRD patterns of Cu-6Ni-6Sn alloy under different excitation currents: (**a**) full-range patterns, (**b**) magnified view of the main characteristic peak.

**Figure 5:**
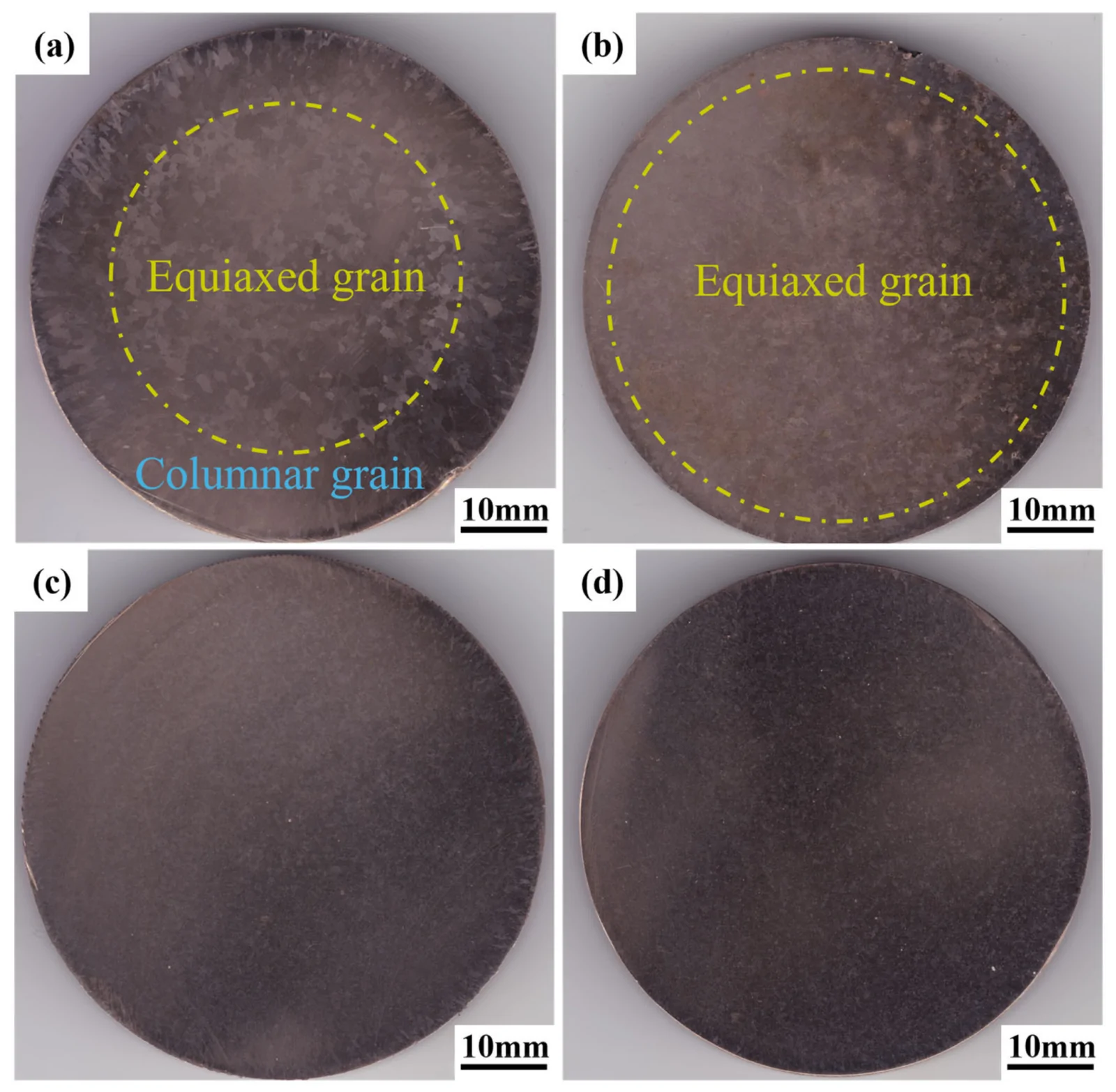
Cross-sectional macrostructures of Cu-6Ni-6Sn alloy under different excitation currents: (**a**) 0 A, (**b**) 40 A, (**c**) 80 A, (**d**) 120 A.

Figure 4 presents the XRD patterns of as-cast Cu-6Ni-Sn alloys subjected to RMF with different excitation currents. As shown in Figure 4a, the excitation current intensity has no obvious influence on the phase constitution of the experimental alloy. All samples exhibit only the face-centered cubic (FCC) α-Cu and the orthorhombic γ-(Cu, Ni)_3_Sn phases with peak positions matching the reference patterns in the ICDD PDF-4+ database (PDF#01-071-3761 for α-Cu, PDF#04-004-6308 for γ-(Cu,Ni)_3_Sn). Furthermore, the full width at half maximum (FWHM) of the α-Cu (111) reflection peak increases monotonically with increasing excitation current from 0 A to 120 A, as highlighted in the enlarged view in Figure 4b. This monotonic broadening qualitatively suggests that the RMF promotes grain refinement. However, it should be noted that the significant intensity variations among the (111) peaks of different samples (e.g., the 40 A and 80 A specimens show higher intensities and sharper peak shapes) may be influenced by preferred orientation (texture) arising from the solidification conditions. Although texture can affect peak intensity, its influence on the FWHM is generally secondary when the peak profile remains symmetric. More importantly, the observed FWHM trend is fully consistent with the macroscopic grain morphology observations (Figure 5), which unambiguously show a progressive reduction in average grain size with increasing excitation current. Therefore, while the absolute crystallite size calculated from the Scherrer equation using a single peak would be unreliable, the relative broadening trend of the (111) peak still provides supportive qualitative evidence for grain refinement.

Unit cell refinement based on α-Cu diffraction peaks was carried out to calculate lattice parameters, and the detailed data are summarized in Table 4. The lattice constant of the sample without RMF (0 A) reaches 3.6480 Å, which is markedly larger than that of RMF-treated specimens. With the increase in excitation current from 0 A to 120 A, the lattice constant gradually decreases to 3.6388 Å at 120 A.

Sn has a larger atomic radius than Cu. Severe Sn macro-segregation exists in the as-cast alloy without RMF, enabling more Sn atoms to dissolve into the α-Cu matrix and induce lattice expansion. The melt convection driven by the rotating magnetic field homogenizes Sn distribution, reduces the solid solubility of Sn in the Cu matrix, and relieves lattice distortion. Higher excitation current enhances the mixing effect of the melt, further promoting element homogenization.

The macrostructures of the Cu-6Ni-6Sn alloy ingot cross-sections under different excitation currents are shown in Figure 5. While the 40 A, 80 A, and 120 A samples all exhibit equiaxed grain structures that may appear visually similar at low magnification, quantitative image analysis reveals a progressive and statistically significant refinement. Without excitation current (0 A), the alloy displays coarse columnar grains, primarily due to the growth of grains in the direction opposite to heat dissipation, resulting in a coarse structure from the periphery to the center. The columnar grains exhibit a major axis of approximately 1700 μm and a minor axis of around 500 μm, corresponding to an aspect ratio of 3.4:1, with their occupied area accounting for 30% of the entire cross-section. When the excitation current increases to 40 A and 80 A, the solidification structure refines significantly, and the average grain size in the central equiaxed zone decreases. However, some columnar grains remain at the edges, as seen in Figure 5b,c. At an excitation current of 120 A, the application of RMF further refines the grains, with the average grain size continuing to decrease. No columnar grains are observed in the cross-section, and the entire macrostructure consists of uniform, fine equiaxed grains.

**Figure 6:**
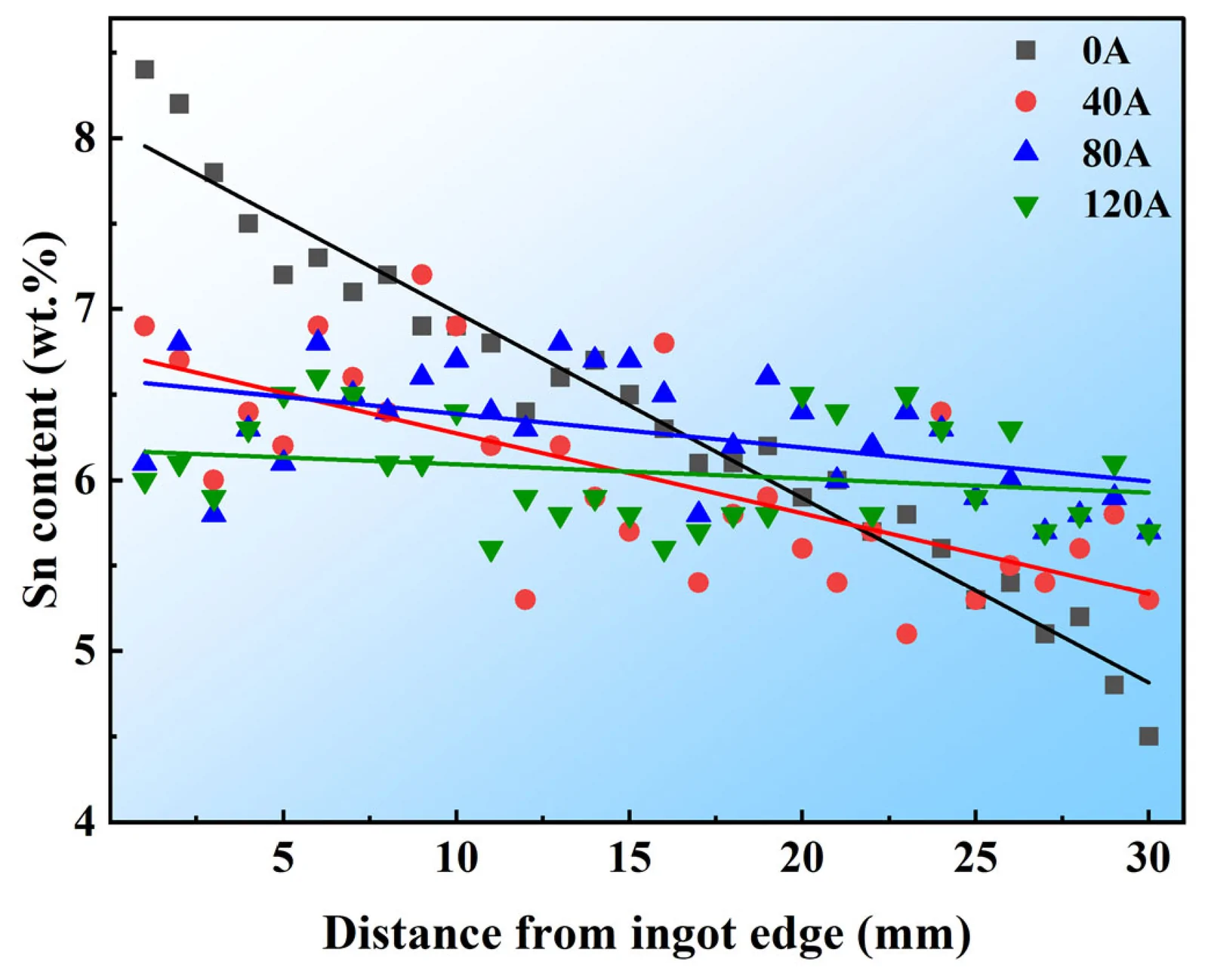
Sn content distribution on the cross-section of Cu-6Ni-6Sn alloy ingot.

The Sn concentration profiles along the radial direction under different excitation currents are presented in Figure 6. To quantitatively evaluate the segregation trend, the data points for each condition were fitted with a linear regression model. The fitting results show a systematic decrease in the absolute slope with increasing excitation current, from −0.108 wt.%/mm at 0 A to −0.008 wt.%/mm at 120 A, while the corresponding R^2^ values decrease from 0.960 to 0.021. This monotonic reduction quantitatively confirms that the RMF progressively suppresses Sn macro-segregation, with the near-zero slope and R^2^ at 120 A indicating an essentially uniform Sn distribution across the entire cross-section. EDS is a semi-quantitative detection method with an inherent measurement error of ±0.2 wt.%. However, the overall radial distribution trend of Sn revealed by the experimental data is reliable and statistically valid.

### 3.2. Microstructure Evolution of Alloy

**Figure 7:**
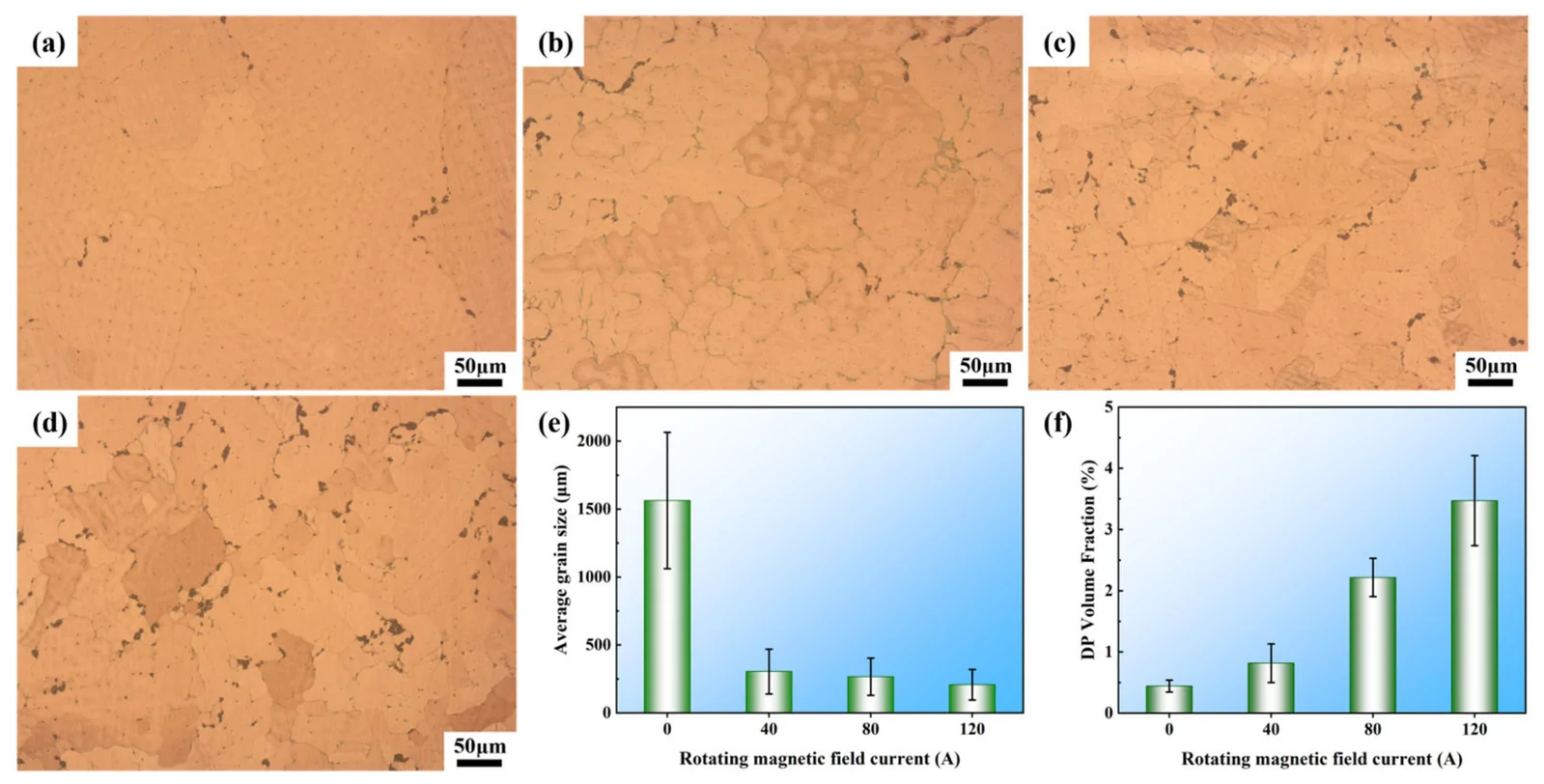
Metallographic structure, grain size and volume fraction of DP phase of the alloy under different excitation currents: (**a**) 0 A, (**b**) 40 A, (**c**) 80 A, (**d**) 120 A, (**e**) average grain size, (**f**) volume fraction of DP phase.

The microstructure, grain size and volume fraction of the discontinuous precipitation (DP) phase of Cu-6Ni-6Sn alloy under different excitation currents are shown in Figure 7. Without the RMF (current 0 A), the microstructure of the alloy ingot is dominated by coarse columnar crystals, and the grain size is not uniform. The average grain size is 1563 ± 502 μm because the melt solidification rate is fast in the traditional casting process, the heat transfer is uneven, the grain growth is not constrained, and the DP phase accounts for a small proportion, as shown in Figure 7a. When the current is 40 A, the number of columnar crystals decreases, the number of equiaxed crystals increases, the average grain size decreases to 303 ± 165 μm, the segregation of Sn element improves, and the proportion of DP phase increases to 1%. When the current is 80 A, the microstructure is dominated by fine equiaxed grains, the grain size is uniform, and the average grain size is 266 ± 137 μm. The content of the Sn element in the central region of the ingot is further increased, and the black DP phase is increased to 2.3%. Because the electromagnetic force generated by the RMF under this current is moderate, it can fully drive the melt to rotate, break the coarse columnar grains, and promote the increase in nucleation cores and inhibit grain growth. When the current increases to 120 A, the average grain size is 207 ± 112 μm and the proportion of black DP phase is 3.5%, as shown in Figure 7f. It shows that the Cu-6Ni-6Sn alloy can obviously refine the grain and improve the segregation of the Sn element under the action of RMF.

### 3.3. Mechanical Properties

**Figure 8:**
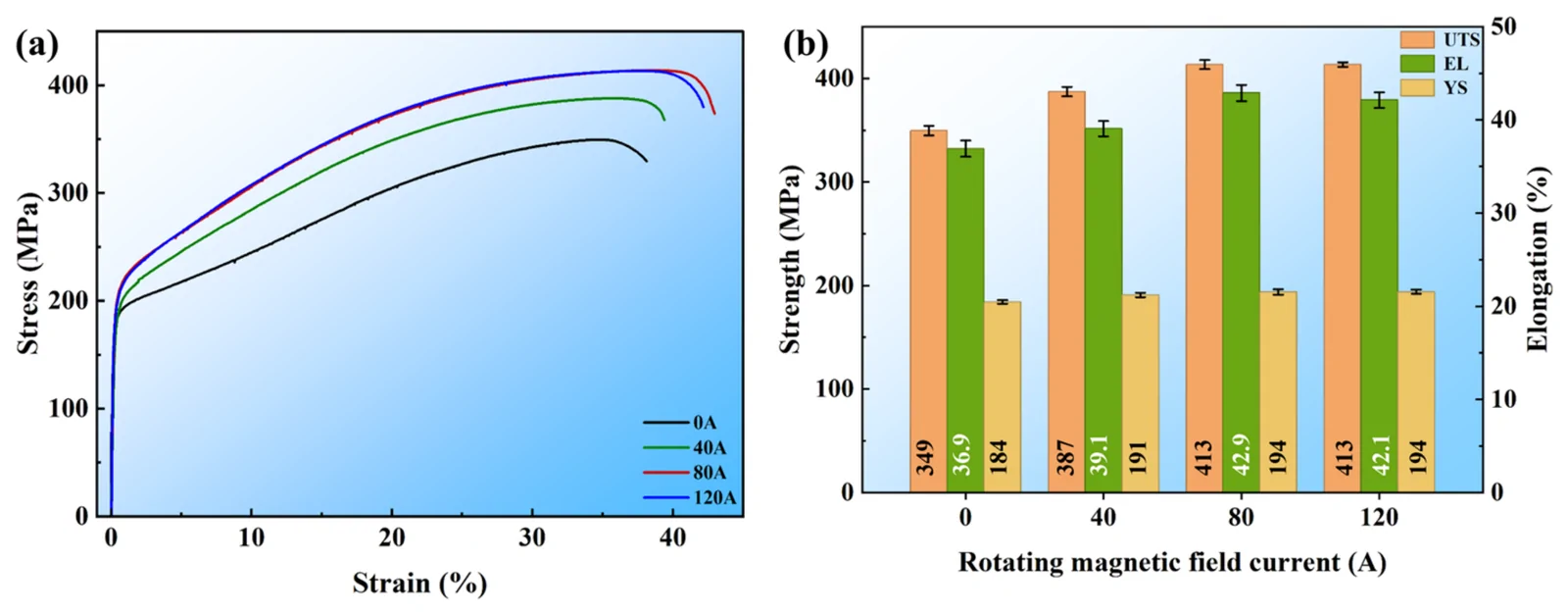
The tensile curves and mechanical property comparison under different excitation currents: (**a**) engineering stress–strain curves, (**b**) mechanical property comparison.

Figure 8 shows the effect of the RMF on the mechanical properties of the Cu-6Ni-6Sn alloy. The application of the RMF significantly enhances both tensile strength and elongation, with mechanical properties initially increasing with higher magnetic field currents before stabilizing. Without the RMF, the tensile strength is 349 ± 4 MPa and the elongation is 36.9 ± 0.8%, indicating relatively poor mechanical performance, mainly due to coarse grains and elemental segregation. At a magnetic field current of 40 A, the tensile strength increases to 387 ± 4 MPa and the elongation reaches 39.0 ± 0.8%. This improvement results from the molten metal’s rotation under the Lorentz force during solidification, which breaks dendrites, refines grains, and promotes a more uniform distribution of Sn, preventing inverse segregation. At 80 A, the alloy shows optimal mechanical properties, with tensile strength reaching 413 ± 4 MPa and elongation increasing to 42.8 ± 0.9%. At 120 A, the tensile strength slightly decreases to 413 ± 2 MPa and the elongation to 42.1 ± 0.8%. Despite these slight reductions, both values remain significantly higher than those of the control group and the 40 A condition. It is noteworthy that the mechanical properties at 80 A and 120 A are very similar, with tensile strengths of 413 ± 4 MPa and 413 ± 2 MPa, respectively, and elongations of 42.8 ± 0.9% and 42.1 ± 0.8%. This suggests that the beneficial effect of RMF on mechanical properties reaches saturation at an excitation current of approximately 80 A under the present conditions.

## 4. Discussion

### 4.1. Mechanism of RMF

#### 4.1.1. Refinement Mechanism

**Figure 9:**
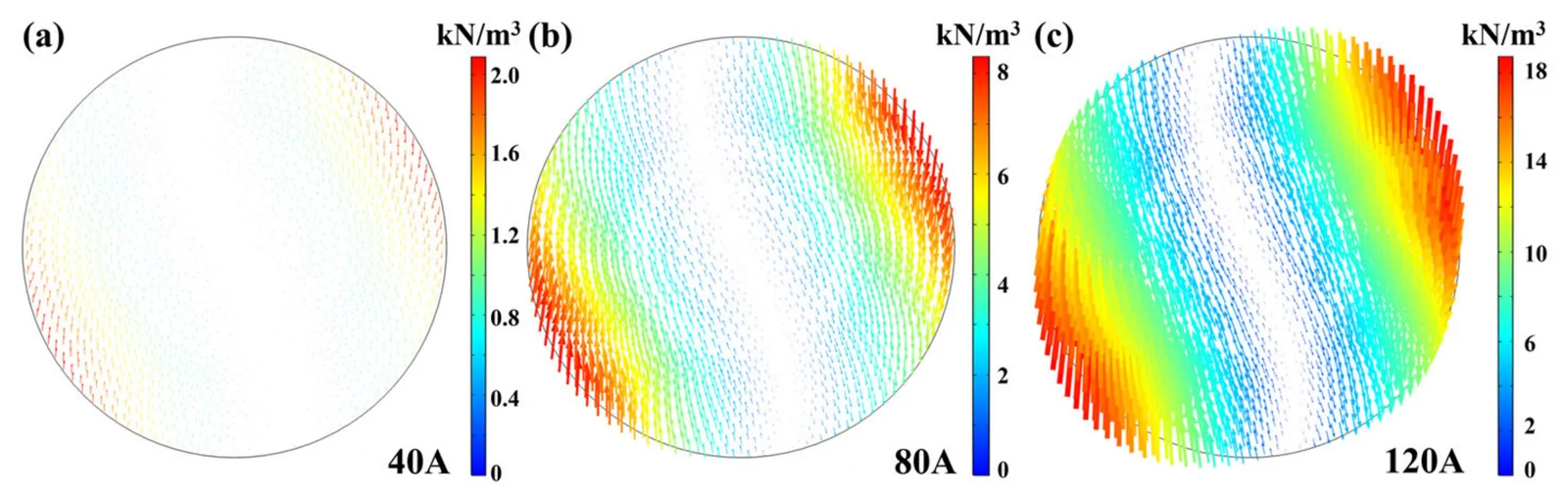
Effect of excitation current on Lorentz force. The arrows represent the direction of the Lorentz force vector, and the color denotes the magnitude of the Lorentz force: (**a**) 40 A, (**b**) 80 A, (**c**) 120 A.

To investigate the flow field distribution in molten copper, numerical simulations were carried out on the alloy solidification process under RMF with different excitation currents. To ensure a steady-state magnetic field, the central cross-section located 160 mm above the ingot bottom was analyzed after the magnetic field had been applied for 100 ms. Figure 9 presents the magnetic field intensity distribution under different excitation currents. As shown in Figure 9a–c, the Lorentz force increases with excitation current and exhibits an edge-high, center-low distribution. The current magnitude exerts a pronounced influence on the Lorentz force. Specifically, its maximum value is approximately 2 kN/m^3^ at 40 A, 8 kN/m^3^ at 80 A and around 18 kN/m^3^ at 120 A.

**Figure 10:**
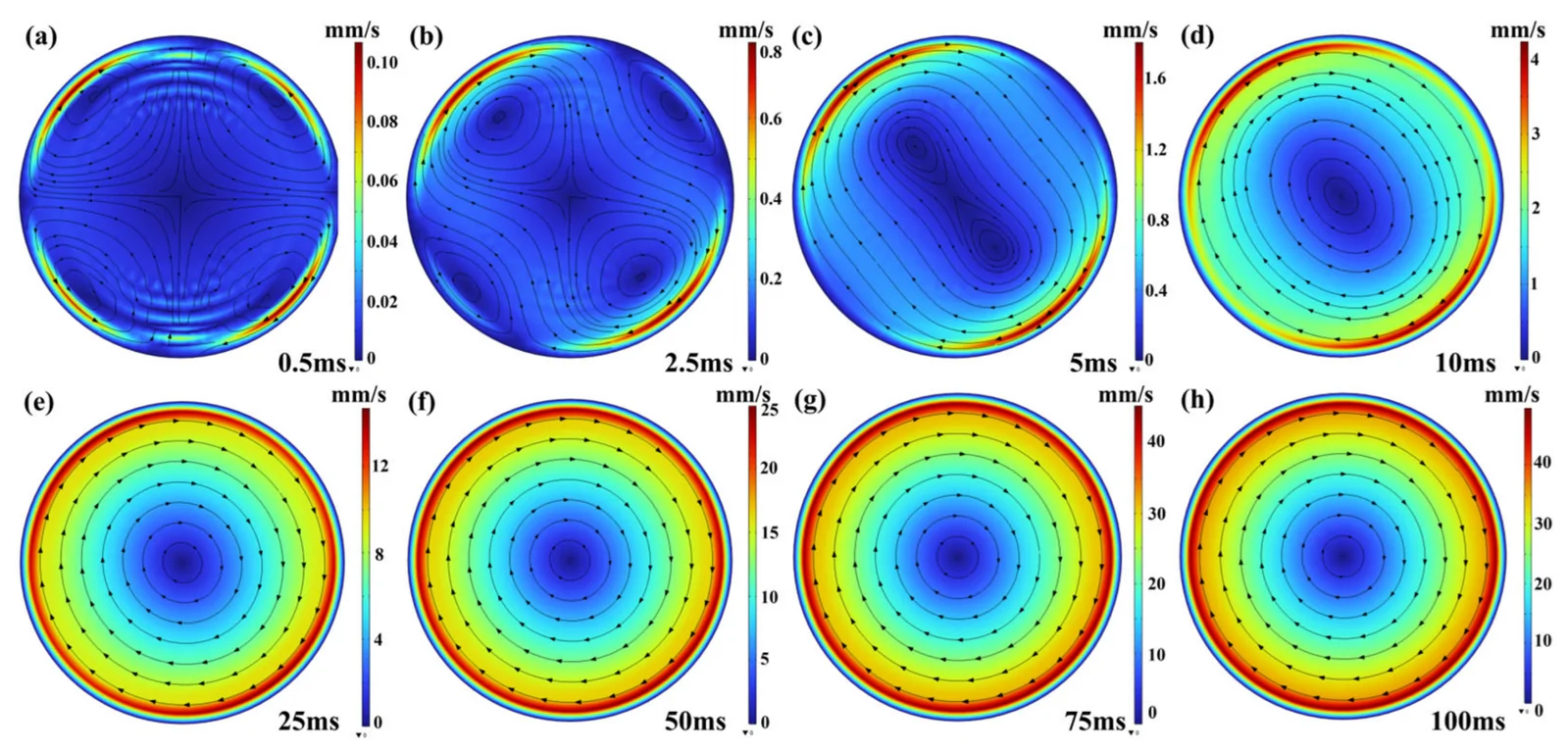
Velocity distribution on the cross-section at different time instants. Black arrows denote flow direction, and color contour shows velocity magnitude: (**a**) 0.5 ms, (**b**) 2.5 ms, (**c**) 5 ms, (**d**) 10 ms, (**e**) 25 ms, (**f**) 50 ms, (**g**) 75 ms, (**h**) 100 ms.

Figure 10 shows the velocity field distribution on the cross-section at different time instants under an excitation current of 80 A. The non-uniform Lorentz force distribution induces rotational motion within the molten copper. Under the effect of the Lorentz force, the melt develops intense forced convection, which effectively refines the solidified grains. It can be seen from Figure 10a that at 0.5 ms, shortly after the magnetic field is applied, the induced current concentrates on the melt surface due to the skin effect, forming four circulating flows inside the melt. At 2.5 ms (Figure 10b), under the action of the Lorentz force, two diagonal circulations strengthen while the other two are compressed. At 5 ms (Figure 10c), the two dominant circulations nearly merge into a single flow, and the initially suppressed circulations disappear. As shown in Figure 10d,e, the flow fully merges into one circulation by 10 ms and the flow pattern becomes essentially stable at 25 ms. Thereafter, the circulation structure remains unchanged over time, while the flow velocity continues to rise. The velocity field eventually reaches a steady state at 100 ms, as illustrated in Figure 10f–h.

**Figure 11:**
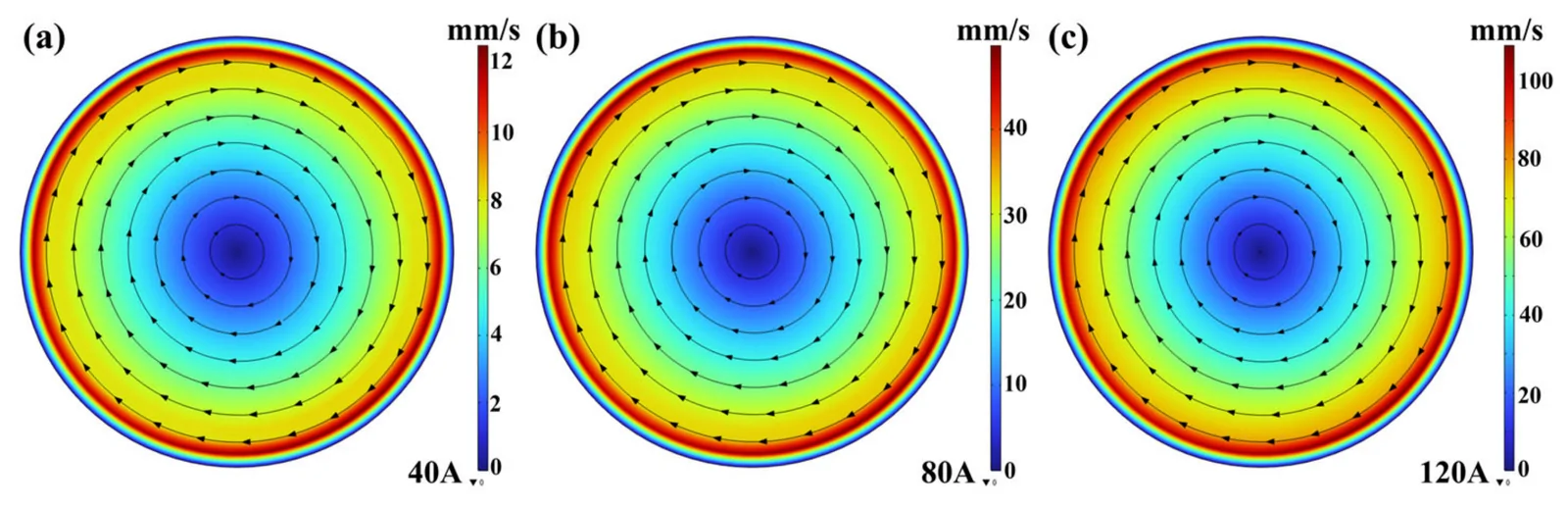
Effect of current intensity on stirring velocity. Black arrows denote flow direction, and color contour shows velocity magnitude: (**a**) 40 A, (**b**) 80 A, (**c**) 120 A.

Figure 11 presents the velocity field distribution at 100 ms under different excitation currents. All cases exhibit an identical flow morphology after stabilization. The main difference lies in flow velocity, which increases with excitation current. A higher excitation current generates a larger Lorentz force, accelerating melt stirring and thus raising flow velocity over the same duration. The variation trend of flow velocity with current is consistent with that of the Lorentz force. As the excitation current rises, the maximum flow velocity increases markedly. When the current grows from 40 A to 120 A, the maximum flow velocity at 100 ms rises from 12.8 mm/s to 107.1 mm/s.

#### 4.1.2. Changes in the Degree of Undercooling

**Figure 12:**
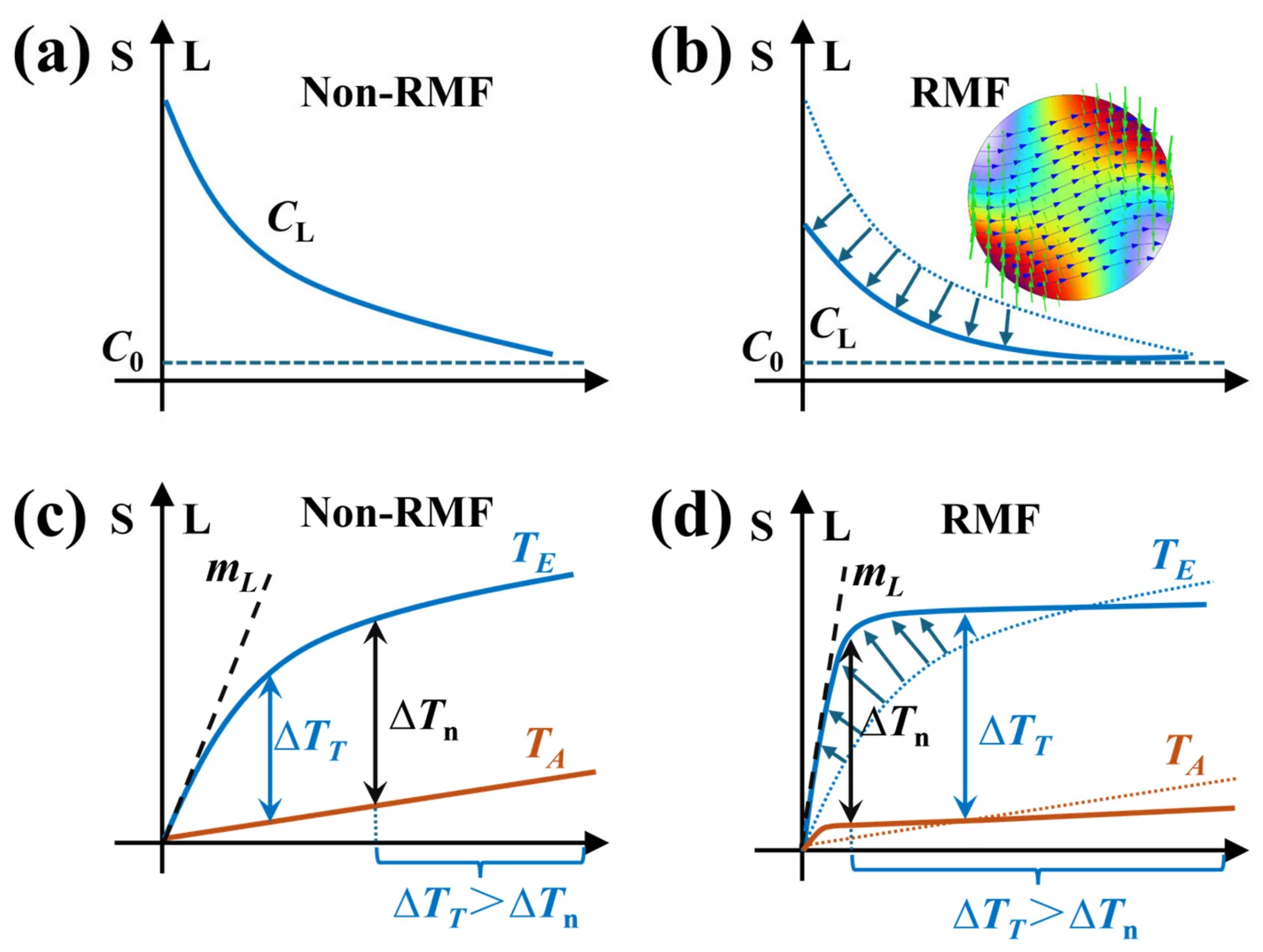
Schematic diagram of constitutional undercooling and thermal undercooling: (**a**,**c**) without RMF, (**b**,**d**) with RMF.

During alloy solidification under RMF, constitutional undercooling (Δ*T*_CS_, ℃) and thermal undercooling (ΔT_T_, ℃) are the dominant factors governing grain refinement. The constitutional undercooling at the solid–liquid interface front can be expressed as [25,26]

(14) $$\Delta T_{CS}=m_{L}C_{0}\frac{\left(1-k_{0})I_{V_{3D}}(P_{eC}\right)}{1-(1-k_{0})I_{V_{3D}}(P_{eC})}$$

(15) $$I_{V_{3D}}(P_{eC})=\frac{C_{L}-C_{0}}{C_{L}(1-k_{0})}$$

where *m_L_* is the slope of the liquidus line, *C*_0_ is the initial alloy composition and *k*_0_ is the equilibrium partition coefficient of the solute. $I_{V_{3D}}(P_{eC})$ is the Ivantsov function related to the Péclet number, and *C_L_* is the solute concentration ahead of the solid–liquid interface. Essentially, constitutional undercooling originates from solute accumulation caused by solute redistribution at the solidification front. The solute concentration at the solidification front and the growth restriction factor can be calculated using the following formulae [27,28]:

(16) $$C_{L}=\frac{C_{0}}{k_{0}+(1-k_{0})\exp(-\frac{R\delta }{D})}$$

(17) $$Q=m_{L}C_{0}(k-1)$$

where *R* is the interface growth rate, *D* is the liquid solute diffusion coefficient, *δ* is the solute diffusion boundary layer thickness and *k* is the partition coefficient. Without the RMF, a thick solute accumulation layer forms ahead of the solid–liquid interface, resulting in a relatively high solute concentration *C_L_* at the solidification front, as shown in Figure 12a. The forced convection induced by the RMF enhances mass and heat transfer [24], which reduces *δ*, solute accumulation and the local constitutional undercooling Δ*T_CS_*. As presented in Figure 12b, dendrite growth is inhibited. Meanwhile, forced convection fractures dendrites and generates a large number of nucleation sites. Uniform undercooling promotes multi-site nucleation. The remelting effect slows the growth of nucleation sites, increases the liquidus slope *m_L_*, and further raises the growth restriction factor *Q*, thus greatly improving the nucleation rate. The thermal undercooling ahead of the solid–liquid interface can be calculated using the following formula [25]:

(18) $$\Delta T_{T}=T_{E}-T_{A}$$

where *T*_E_ is the equilibrium liquidus temperature (°C) and *T*_A_ is the actual melt temperature (°C). Nucleation occurs only when the thermal undercooling Δ*T_T_* ahead of the solid–liquid interface exceeds the critical undercooling Δ*T*n (°C) [29,30]. After the RMF is applied, the rotation of the melt homogenizes the temperature field, raises *T_E_*, and thereby increases Δ*T_T_*, consequently facilitating nucleation, as shown in Figure 12c,d.

**Figure 13:**
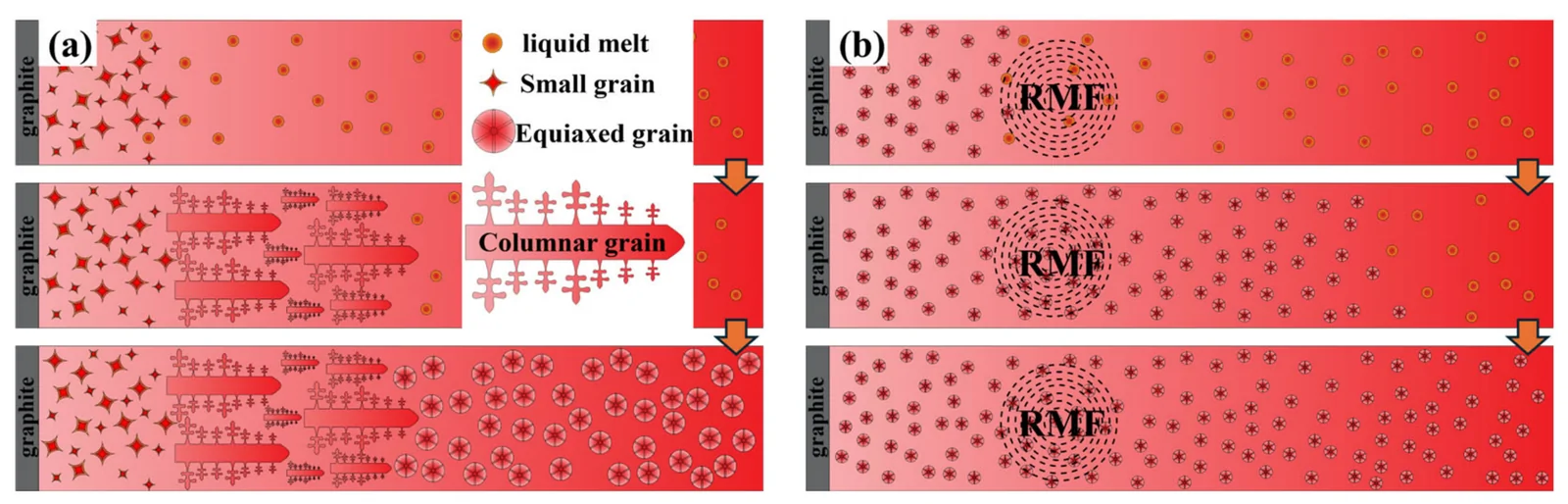
Schematic illustration of grain growth: (**a**) without RMF, (**b**) with RMF.

Figure 13 shows the grain growth morphology. Without the RMF, the ingot exhibits a typical solidification structure consisting of a surface fine-grain zone, a columnar grain zone, and a central equiaxed grain zone. The grains in the central equiaxed grain zone are relatively coarse, as shown in Figure 13a. When the RMF is applied, numerous fine grains distribute uniformly in the melt at the early solidification stage, and their growth is suppressed. At the late stage of solidification, these densely distributed fine grains grow simultaneously and mutually inhibit coarsening. Consequently, a uniform and refined equiaxed grain structure is obtained, as shown in Figure 13b.

#### 4.1.3. Suppression of Sn Segregation

Based on the non-equilibrium solidification characteristics of the Cu-6Ni-6Sn alloy, without the RMF, Sn-rich melt migrates toward the ingot surface along columnar grain channels under solidification shrinkage, thereby inducing severe inverse segregation. The application of RMF generates pervasive forced convection within the molten pool, which homogenizes the spatial distribution of Sn solute, reduces constitutional undercooling, and restricts localized Sn enrichment. Magnetic field-driven grain refinement and the increased fraction of equiaxed grains further suppress Sn segregation. In addition, melt flow continuously fractures dendrite arms during solidification, further enhancing solute uniformity throughout the ingot.

### 4.2. Strengthening Mechanism

For the Cu-6Ni-6Sn alloy solidified under the RMF, grain boundary strengthening and precipitation strengthening dominate the overall strengthening mechanisms. The increase in yield strength caused by grain refinement can be quantified using the Hall–Petch equation [31]:

(19) $$\Delta \sigma_{GBS}=k_{H}d^{-\frac{1}{2}}$$

where Δ*σ_GBS_* is the yield strength increment caused by grain refinement, k_H_ is a material constant and *d* represents the average grain size. For copper alloys, k_H_ = 0.18 MPa·m^1/2^. This k_H_ value is quoted from Ref. [31] for Cu-15Ni-8Sn alloy, and its minor uncertainty does not change the main strengthening trend. By substituting the grain sizes of the alloy obtained under different excitation currents into the formula, the data in Table 5 are obtained, while the remaining increments in yield strength are attributed to precipitation strengthening. Specifically, as shown in Table 5, the discrepancy between theoretical and experimental strength increments exhibits a clear current-dependent trend. At the low excitation current of 40 A, the theoretical grain boundary strengthening increment (6 MPa) is very close to the experimental yield strength increment (7 MPa). This is because the weak forced convection only achieves limited grain refinement, and the suppression effect on Sn microsegregation is not prominent, so the total strengthening is dominated by grain boundary strengthening. When the current rises to 80 A and 120 A, the experimental strength increments both reach 10 MPa, which are significantly higher than the theoretical values (7 MPa and 8 MPa, respectively). This extra strength contribution originates from the intensified segregation inhibition: strong melt flow homogenizes the distribution of Sn solute atoms and intermetallic precipitates, reduces local stress concentration at grain boundaries, and further enhances the precipitation strengthening effect, which is not included in the pure grain boundary strengthening calculation of the Hall–Petch model. The RMF effectively suppresses dendritic and inverse segregation during alloy solidification and homogenizes the distribution of discontinuous precipitates, thereby providing an additional contribution to yield strength. The fine equiaxed grains reduce grain boundary defects, improve grain boundary bonding strength, and homogenize elemental distribution, which prevents local stress concentration. These results demonstrate that grain refinement and segregation inhibition constitute the core mechanisms through which the RMF enhances the mechanical properties of the Cu-6Ni-6Sn alloy, synergistically improving both its tensile strength and plasticity.

The extent of grain refinement observed in this study (from 1563 μm to 207 μm) is comparable to or even more pronounced than that reported for Cu-12Sn-2Ni alloys under electromagnetic stirring, where the average grain size decreased from 587 μm to 319 μm [32]. Similarly, the simultaneous improvement in tensile strength (from 349 MPa to 413 MPa) and elongation (from 36.9% to 42.8%) aligns with the general trend reported for Cu-Sn alloys processed under RMF, where strength and ductility increases of 11.1% and 78% were achieved, respectively. These comparisons confirm that RMF is an effective and generalizable approach for enhancing the mechanical properties of Cu-Ni-Sn alloys through solidification structure control.

**Figure 14:**
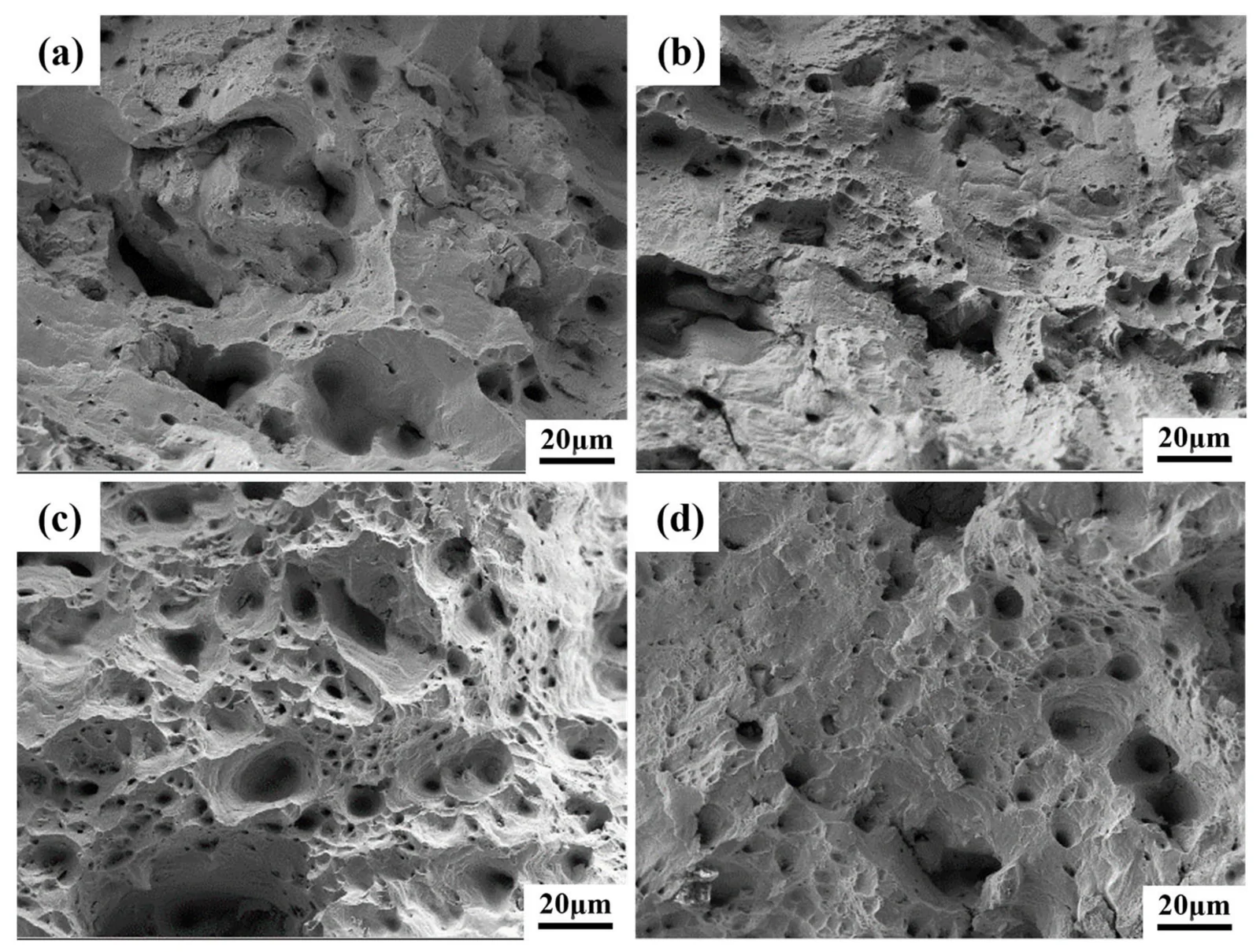
Tensile fracture morphologies under different excitation currents: (**a**) 0 A, (**b**) 40 A, (**c**) 80 A, (**d**) 120 A.

Figure 14 presents the tensile fracture morphologies of the Cu-6Ni-6Sn alloy under different excitation currents. Without a magnetic field, the fracture surface displays a few shallow dimples, numerous tearing ridges, and a few cleavage planes. At an excitation current of 40 A, the number of dimples increases, and the tearing ridges almost disappear. As the excitation current rises to 80 A and 120 A, many equiaxed dimples appear on the fracture surface, indicating a typical ductile fracture characteristic.

## 5. Conclusions

In summary, this study systematically investigated the effects and underlying mechanisms of RMF on the solidification microstructure and mechanical properties of the Cu-6Ni-6Sn alloy through a combination of experimental characterization and finite element simulations. The main conclusions are summarized as follows:

(1) The RMF exerts a pronounced grain refinement effect on the Cu-6Ni-6Sn alloy. The average grain size decreases from 1563 ± 302 μm without RMF treatment to 266 ± 137 μm under RMF at 80 A and 20 Hz.
(2) The RMF promotes the complete transition from columnar grains to equiaxed grains. Melt stirring induced by RMF effectively suppresses the reverse segregation and microsegregation of Sn, resulting in a more homogeneous composition. The homogenized microstructure provides a favorable basis for subsequent plastic processing and improved mechanical properties.
(3) The RMF generated by alternating current induces a spatially nonuniform Lorentz force with rotational characteristics in the copper alloy melt. This force drives melt rotation and strong forced convection during solidification. With increasing excitation current, both the Lorentz force and the melt stirring velocity increase significantly, thereby enhancing melt flow regulation.
(4) The RMF effectively enhances the mechanical properties of the alloy through grain refinement strengthening. Combined with the homogeneous distribution of Sn solute, this microstructural refinement significantly improves the overall microstructure and alleviates localized stress concentrations. Under RMF of 80 A and 20 Hz, the alloy achieves simultaneous improvement in both strength and ductility compared with the RMF-free ingot: the ultimate tensile strength increases from 349 ± 4 MPa to 413 ± 4 MPa, while the elongation rises from 36.9 ± 0.8% to 42.8 ± 0.9%.
(5) Regarding industrial scale-up to large-diameter ingots (>100 mm), the current RMF process still has certain limitations. The electromagnetic skin effect causes significant field attenuation from the ingot surface to the core, weakening melt convection and microstructure regulation efficiency in the central region, which cannot be compensated only by increasing the excitation current. Follow-up work will focus on optimizing excitation frequency, multi-coil configuration and cooling matching to achieve uniform treatment of large-size ingots.

## Figures and Tables

**Table 1:** Elemental composition of Cu-6Ni-6Sn alloy.

Samples	Nominal	Ni	Sn	Cu
0 A	Cu-6Ni-6Sn	6.15	5.92	Balance
40 A	Cu-6Ni-6Sn	6.10	5.88	Balance
80 A	Cu-6Ni-6Sn	6.07	5.80	Balance
120 A	Cu-6Ni-6Sn	6.13	5.84	Balance

**Table 2:** Primary physical parameters adopted in the numerical model.

	Molten Copper	Coil	Iron Core	Graphite	Air
Electrical conductivity S/m	1.75 × 10^6^	2.71 × 10^7^	1.12 × 10^7^	3.00 × 10^3^	0
Relative magnetic permeability	1.14	1.14	1000	1	1

**Table 3:** Primary thermophysical parameters of the Cu-6Ni-6Sn alloy.

Thermophysical Properties	Value	Unit
Dynamic viscosity	0.004509	Pa·s
Specific heat	380	J/(kg·K)
Thermal conductivity	55	W/(m·K)
Density	8600	kg/m^3^

**Table 4:** Lattice parameters of α-Cu matrix refined from XRD patterns.

Excitation Current/A	Lattice Constant a/Å
0	3.6480
40	3.6410
80	3.6402
120	3.6388

**Table 5:** Difference between theoretical and experimental strength values.

Excitation Current	Theoretical Value	Theoretical Difference	Experimental Yield Strength	Experimental Difference
0 A	4	-	184	-
40 A	10	6	191	7
80 A	11	7	194	10
120 A	12	8	194	10

## Data Availability

The original contributions presented in this study are included in the article. Further inquiries can be directed to the corresponding author.

## References

[1] Zuo P., Zhang Z., Qi X., Liu Z., Xia J., Wu J., Li X., Zhang B. (2023). Effects of nanoscale Sn segregation on corrosion behavior of laser powder bed fusion Cu-15Ni-8Sn alloy. J. Mater. Sci. Technol..

[2] Zuo P., Ju H., Liu Z., Zhang B., Zhang Z., Liu M., He S., Luo X., Li Y., Peng B. (2025). Influence of heat treatment on the corrosion and tribo-corrosion resistance of LPBF additively manufactured Cu-15Ni-8Sn alloy. Corros. Sci..

[3] Guo L., Zuo P., Zhang Z., Zhang Q., Zhao M., Hou X., Wu J., Zhang B. (2023). A Review of Cu-Ni-Sn Alloys: Processing, Microstructure, Properties, and Developing Trends. Materials.

[4] Sankar B., Vinay C., Vishnu J., Shankar K.V., Krishna G.P.G., Govind V., Jayakrishna A.J. (2022). Focused Review on Cu-Ni-Sn Spinodal Alloys: From Casting to Additive Manufacturing. Met. Mater. Int..

[5] Xie Z., Li Z., Tang D., Wang Z., Cui H., Kong C., Yu H. (2024). Effects of Cryorolling, Room Temperature Rolling, and Aging Treatment on the Mechanical, Electrical, and Wear Properties of a Cu-6Ni-6Sn Alloy. Metall. Mater. Trans. A.

[6] Zhang K., Zhang L., Shu C., Fan W., Li S., Yuan X., Zhao J., Wang Y., Wang P. (2022). Effect of Different Mo Addition on Microstructure and Mechanical Properties of Cu-15Ni-8Sn Alloy. Materials.

[7] Yang W., Zhou Y., Yang R., Yang S., Zhou F., Song K., Feng J., Jiang H., Zhang X., Du J. (2024). Effect mechanism of Nb addition on grain refinement and inhibition of discontinuous precipitation of Cu-15Ni-8Sn alloy. J. Mater. Res. Technol..

[8] Ouyang Y., Gan X.-P., Zhang S.-Z., Li Z., Zhou K.-C., Jiang Y.-X., Zhang X.-W. (2017). Age-hardening behavior and microstructure of Cu-15Ni-8Sn-0.3Nb alloy prepared by powder metallurgy and hot extrusion. Trans. Nonferrous Met. Soc. China.

[9] Zhou F., Zhou Y., Yang R., Song K., Lu L., Zhang Y., Yang S., Yue P., Meng X., Chen J. (2024). Mn-Induced microstructural stabilization and mechanical strengthening in Cu-Ni-Sn alloys: A mechanistic exploration. Mater. Sci. Eng. A.

[10] Xie S., Zhao C., Dong H., Zhong R., Li D., Ke Y., Zhang W. (2025). Effect of Si addition on the microstructure evolution of cast Cu-15Ni-8Sn alloys during solidification. J. Alloys Compd..

[11] Lu S., Du S., Wang X., Ding Z., Liu X., Li Z. (2024). Microstructure and mechanical properties of Cu-Ni-Sn alloy regulated by trace rare earth element Y. Mater. Charact..

[12] Zhang J.J., Liu Z.Y., Guo Z.K., Liu S.C., Dong Y., Fu Y., Jie J.C., Zhang D.L. (2022). Effects of Zr on microstructural evolution and mechanical property of Cu-15Ni-8Sn alloy. Mater. Sci. Eng. A-Struct. Mater. Prop. Microstruct. Process..

[13] Lu S., Du S., Li Z., Liu X., Wang X., Ding Z. (2025). Effect of Zr addition on microstructure, mechanical and tribological properties of Cu-Ni-Sn alloy fabricated by spark plasma sintering. J. Alloys Compd..

[14] Luo L., Luo L., Ritchie R.O., Su Y., Wang B., Wang L., Chen R., Guo J., Fu H. (2021). Optimizing the microstructures and mechanical properties of Al-Cu-based alloys with large solidification intervals by coupling travelling magnetic fields with sequential solidification. J. Mater. Sci. Technol..

[15] Liu J., Jie J., Wang X., Lou S., Peng B., Li T. (2022). Formation Mechanism of Three-Layer Solidified Structure in Commercial Purity Al During Solidification With a Forced Flow Field Induced by Pulsed Magnetic Field. Metall. Mater. Trans. A.

[16] Zhang H., Wu M., Rodrigues C.M.G., Ludwig A., Kharicha A., Rónaföldi A., Roósz A., Veres Z., Svéda M. (2022). Dendrite fragmentation mechanism under forced convection condition by rotating magnetic field during unidirectional solidification of AlSi7 alloy. Acta Mater..

[17] Liu S., Wang X., Jie J., Wu X., Xu S., Li X., Li T. (2023). Effect of rotating magnetic field on NiAl(Si) phase evolution and mechanical properties of special nickel brass. J. Alloys Compd..

[18] El-Taher A.M., Ali H.E., Algarni H. (2024). Investigating the influence of rotating magnetic field (RMF) on intermetallic compound crystallization, thermal and mechanical properties in lead-free Sn-Ag-Cu solder alloys during solidification. Mater. Today Commun..

[19] Li G., Jie J., Liu J., Wang M., Hang Z., Liu J., Guo Z., Li T. (2025). Influence of rotating magnetic field on microstructure and strength-ductility synergy in wide liquidus-solidus interval phosphor bronze. J. Mater. Sci. Technol..

[20] Zhao S., Wei G.W. (2004). High-order FDTD methods via derivative matching for Maxwell’s equations with material interfaces. J. Comput. Phys..

[21] Liang Z., Fan W., Wang P., Wang Y., Zhang K., Zhao J., Peng L. (2021). Revealing the Effect of Phase Composition and Transformation on the Mechanical Properties of a Cu-6Ni-6Sn-0.6Si Alloy. Materials.

[22] Amsden A.A., Harlow F.H. (1970). A simplified MAC technique for incompressible fluid flow calculations. J. Comput. Phys..

[23] Hajjari E., Divandari M., Razavi S.H., Emami S.M., Kamado S. (2011). Estimation of the transient interfacial heat flux between substrate/melt at the initiation of magnesium solidification on aluminum substrates using the lumped capacitance method. Appl. Surf. Sci..

[24] Xu J., Li J., Ma Y., Hu W., Zeng W., Zhang H., Huang J. (2026). Numerical simulation and study of flow and heat transfer performance of direct contact heat exchanger based on sinusoidal alternating magnetic field. Therm. Sci. Eng. Prog..

[25] Pan S., Zhu M. (2010). A three-dimensional sharp interface model for the quantitative simulation of solutal dendritic growth. Acta Mater..

[26] Zhang X., Zhao J., Jiang H., Zhu M. (2012). A three-dimensional cellular automaton model for dendritic growth in multi-component alloys. Acta Mater..

[27] Kurz W., Fisher D., Rappaz M. (2023). Fundamentals of Solidification.

[28] StJohn D., Qian M., Easton M., Cao P. (2011). The Interdependence Theory: The relationship between grain formation and nucleant selection. Acta Mater..

[29] Zhang D., Qiu D., Gibson M.A., Zheng Y., Fraser H.L., StJohn D.H., Easton M.A. (2019). Additive manufacturing of ultrafine-grained high-strength titanium alloys. Nature.

[30] StJohn D., Prasad A., Easton M., Qian M. (2015). The contribution of constitutional supercooling to nucleation and grain formation. Metall. Mater. Trans. A.

[31] Zhang G., Liu S., Chen C., Zhang X., Gan X., Lei Q., Li Z., Zhou K. (2019). Effect of heat treatment on microstructure and mechanical properties of a selective laser melted Cu-15Ni-8Sn alloy. Mater. Sci. Eng. A.

[32] Tian G., Liu J., Peng B., Wang X., Jiang N., Mai Y., Kang H., Li T., Jie J. (2026). Effect of rotating magnetic field on the microstructure and properties of Cu-12Sn-2Ni alloy based on multi-physical field simulation. Mater. Sci. Technol..

